# The clinical course of patients with previous acute and recurrent pericarditis receiving the BNT162b2 vaccine

**DOI:** 10.1016/j.ijcha.2022.101084

**Published:** 2022-07-18

**Authors:** Yishay Wasserstrum, Sofia Nadav, Amitai Segev, Dor Lotan, Dov Freimark, Michael Arad

**Affiliations:** a Chaim Sheba Medical Center, Tel Hashomer, Israel; b Sackler School of Medicine, Tel Aviv University, Tel Aviv, Israel; c St. George School of Medicine, University of London, London, UK; d Division of Cardiology, Columbia University Medical Center, NewYork-Presbyterian Hospital in New York, New York, USA

**Keywords:** Pericarditis, Recurrent pericarditis, Vaccine, COVID19, Adverse Events

## Abstract

**Background:**

Vaccines against SARS-COV2 have been crucial in efforts against COVID19, yet there have been reports of pericarditis following vaccination with mRNA-based vaccines.

**Methods:**

We questioned consecutive patients with a history of acute pericarditis (AP) evaluated in the pericardial disease clinic during 3–11/2020 in a single tertiary center. Patients with significant myocardial involvement or pericarditis secondary to another systemic disease were excluded.

**Results:**

We included 64 patients in the final analysis. Mean age was 53.1 (±18), and 26 (41%) were female. At least 1 recurrence of AP was documented in 47 (73%) cases, 32 (50%) had ≥ 3 recurrences prior to vaccination. AP was considered to be idiopathic\viral in 45 (70%) cases, 20 (31%) cases were post-injury. All patients received at least 2 doses of the vaccine, and 48 patients (75%) received a 3rd dose. Two cases of breakthrough COVID19 infections were documented. Overall, 12 patients (19%) reported any adverse events. Of which, 2 had recurrent pericarditis. There was a trend for a younger age in those patients who had adverse events (median age 45 [IQR 36–61] vs. 60 [38–71], p = 0.08). no other significant difference was seen.

**Conclusion:**

In patients with a history of acute\recurrent pericarditis, the use of BNT162b2 was mostly uneventful, but some mild disease recurrences did occur.

## Highlights

• Pericarditis has been reported following the BNT162b2 vaccine.
• The safety of BNT162b2 in patients with previous pericarditis is unknown.
• Some cases of mild symptoms following BNT162b2 in previous pericarditis were seen.
• Pericarditis recurrence was seen in 2 of 64 patients, who both had a recently active disease.

## 1 Introduction

Acute pericarditis (AP) is a relatively common condition,reported to account for approximately 5% of referrals to Emergency Departments for chest pain, and 0.2% of all cardiovascular in-hospital admissions [1]. While mostly benign, AP may have a relapsing pattern with multiple recurrent episodes of varying severity, that may impair quality of life and lead to the use of chronic corticosteroid or immunomodulatory therapy [2]. These patients present a challenge both in managing the underlying illness, and also in the need to provide support and patient's reassurance regarding risk and potential triggers for recurrent events.

Pericarditis has been reported as a rare event following vaccination, mostly in men receiving smallpox or anthrax vaccines [3].Vaccines against SARS-COV2 have been crucial in efforts against COVID19. Both mRNA-based agents, BNT162b2 (Pfizer\BioNTech) and mRNA-1273 (Moderna) have been reported to cause myocarditis or pericarditis [4], [5], [6]. A personal history of AP was not considered as an exclusion-criteria for participation in these trials. Because immunological stimuli may constitute a trigger to AP or recurrent pericarditis (RP), giving advice on SARS-COV2 vaccine to patients with a history of AP or RP, is challenging.

## 2 Methods

We questioned consecutive patients with a prior history of acute and recurrent pericarditis who were evaluated in the pericardial disease clinic in a single tertiary center in Israel during 3–11/2020. The Pericardial Diseases Clinic is a nationwide referral center for complicated cases of pericarditis.

All patients were aged > 18, and both males and females were recruited. All participants had their first clinic evaluation prior to 11/2020, establishing that AP proceeded administration of BNT162b2. Patients with significant myocardial involvement or pericarditis secondary to a rheumatic, malignant or other systemic disease were excluded. Data was collected via clinic or telephone surveys, and from electronic medical record files.

## 3 Results

Of the 68 patients eligible for inclusion, 64 received the SARS-COV2 vaccine and were included in the final analysis. Mean age at the time of the 1st vaccine dose was 53.1 (±18), and 26 (41%) were female. The majority of patients had at least 1 episode of RP (n = 47, 73%), and approximately half had 3 or more recurrences (n = 32, 50%). AP was considered to be idiopathic\viral in 44 (69%) cases, 19 (30%) cases were post-injury, and the last case was associated with acute Coxiella burnetii infection. Three patients had experienced adverse events (AE) following prior vaccinations: one had an episode of AP following an influenza vaccine, and another had self-limited chest pain after hepatitis B vaccination. Another patient had a history of RP following influenza vaccine.

All patients received at least 2 doses of the vaccine, and 48 patients (75%) received a 3rd booster dose. The 4 patients who did not receive vaccines all had multiple previous episodes of AP recurrences, and were either reluctant to consent due to publications regarding the risk AP occurrence post-vaccine, or advised against vaccination by an external treating physician. Two cases of breakthrough COVID19 infections were documented, 1 in a patient who received all 3 doses, with no pericarditis recurrence. One of these patients was hospitalized in another facility, with no data available on COVID19 severity or course.

During the period of vaccine administration, 30 patients (47%) were receiving some form of regular anti-inflammatory treatment. Ten patients (16%) were treated with oral prednisone, mostly ranging from 5 to 10 mg OD. One patient was receiving high-dose aspirin, and 1 more was receiving ibuprofen. Ten patients (16%) were treated with advanced immunomodulatory therapy, 2 (3%) were on azathioprine, 6 (9%) were on anakinra and 2 (3%) were on rilonacept. All aforementioned treatments were well tolerated during long-term follow-up, with no infectious complications. 24 patients (38%) were receiving colchicine. None of the patients had previous surgical pericardial interventions.

**Fig. 1:**
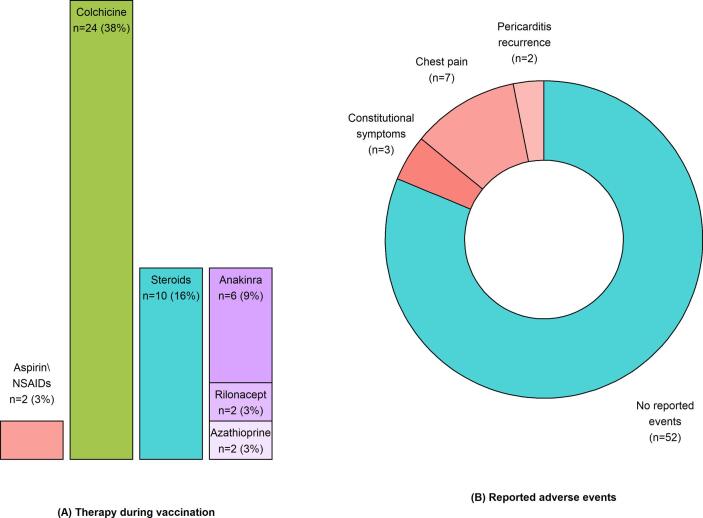
Medical threapy and adverse events.

Overall, 12 patients (19%) reported any AE reported in the 3 weeks following SARS-COV2 vaccine. Nine patients (14%) reported chest pain. Of these, 2 (3%) were diagnosed as AP recurrence, both sought care in a primary-care setting not requiring hospital admission, and began therapy based on typical chest pain, elevated inflammatory markers and a previous history of pericarditis. The other patients who reported chest pain had self-limited symptoms or used over-the-counter analgesics or short-term ibuprofen, and did not seek further medical evaluation. Seven patients (11%) reported constitutional symptoms such as weakness, fatigue, fever or chills (Fig. 1). All cases were managed in as outpatients. There was a trend for a younger age in those patients who had AEs (median age 45 years [IQR 36–61] vs. 60 years [38–71], p = 0.08), and less patients receiving a 3rd dose in the AE group (50% vs. 83%, p = 0.02), no other significant difference was seen.

## 4 Discussion

Patients with a history of pericarditis, especially recurrent disease, are often anxious and fear the possibility of disease recurrence. During the first days of the COVID19 vaccination efforts, almost all of the patients seen in our clinic enquired on safety specifically regarding their cases. This was driven by reports of pericarditis in clinical trials of the mRNA-based vaccines, and by the individual internal conflict created by the co-incidence of these worries and the regulatory pressure to take action in order to get vaccinated and avoid personal sanctions. In the absence of relevant published data, we were unable to give our patients advice beyond out personal expert opinion. Our data, with all the limitations associated with non-randomized single-arm observational data, provides the first piece of evidence regarding the safety of BNT162b2 in this challenging population of patients.

We note that several patients reported some prophylactic use of ibuprofen and\or colchicine for several days before and after vaccination (no reportable data available), which might be a useful mitigating strategy to further reduce risk of AE in these patients. This strategy was not evidence-based, and should be prospectively investigated. Furthermore, as non-mRNA-based SARS-COV2 vaccines have not been associated with either myocarditis or pericarditis, these agents may be useful in patients with history of AP and RP who do not get vaccinated with mRNA-based vaccines due to concerns for recurrence of pericarditis.

In conclusion, in a real-life cohort of patients with a history of acute and recurrent pericarditis, the use of the BNT162b2 vaccine against SARS-COV2 was mostly uneventful, but suffered from minor AEs, including chest pain that was either self-limited or required minimal over-the-counter analgesics, while uncomplicated AP recurrences were seen in 3% of cases.

## 5 Author statement

Dr. Yishay Wasserstrum: Involved in all elements of this study including conceptualization, methodology, validation, execution, formal analysis, supervision, and manuscript preparation and revision.

Dr. Sofia Nadav: Involved in conceptualization, methodology, validation, execution, formal analysis, and manuscript preparation and revision.

Dr. Amitai Segev: Involved in manuscript preparation, data analysis, data presentation, and manuscript revision.

Dr. Dor Lotan: Involved in manuscript preparation, data analysis, and manuscript revision.

Dr. Dov Freimark: Involved in manuscript preparation, data analysis, and manuscript revision.

Dr. Michael Arad: Involved in conceptualization, methodology, validation, execution, formal analysis, supervision, and manuscript preparation and revision.

## Declaration of Competing Interest

The authors declare that they have no known competing financial interests or personal relationships that could have appeared to influence the work reported in this paper.
